# A trial of oral corticosteroids for persistent systemic and airway inflammation in severe asthma

**DOI:** 10.1002/iid3.166

**Published:** 2017-05-04

**Authors:** Keiji Oishi, Tsunahiko Hirano, Ryo Suetake, Syuichiro Ohata, Yoshikazu Yamaji, Kosuke Ito, Nobutaka Edakuni, Kazuto Matsunaga

**Affiliations:** ^1^ Department of Respiratory Medicine and Infectious Disease, Graduate School of Medicine Yamaguchi University Ube Japan; ^2^ Division of Cardiology, Department of Medicine and Clinical Science, Graduate School of Medicine Yamaguchi University Ube Japan

**Keywords:** Asthma, blood eosinophils, exhaled nitric oxide

## Abstract

**Introduction:**

The fraction of exhaled nitric oxide (FeNO) and blood eosinophils, markers of local and systemic eosinophilic inflammation, respectively, are increased in asthmatic patients. Little is known concerning the relationship between the FeNO levels and blood eosinophils in asthmatics.

**Methods:**

Twenty severe asthmatics with persistent FeNO elevation (≥40 ppb) and blood eosinophilia (≥3%) despite maintenance therapy including high‐daily‐dose inhaled corticosteroids were analyzed. We investigated the response of FeNO and blood eosinophils to systemic corticosteroids treatment and the change in Asthma Control Questionnaire (ACQ) according to differences in the response of FeNO and blood eosinophils to steroid.

**Results:**

The changes in blood eosinophils were not correlated with the changes in FeNO levels by systemic steroid treatment (*r* = 0.37, *P* = 0.11). 50% of the subjects showed both ≥20% reductions in FeNO levels and blood eosinophils. There were significant differences in the ACQ score between the steroid response group and poor response group (*P* < 0.005). The group in which both FeNO and blood eosinophils were suppressed fulfilled the change in score of ≥0.5 on the ACQ.

**Conclusions:**

In the patients with severe asthma, responses to systemic corticosteroids were variable in terms of FeNO and blood eosinophils. It was necessary to suppress both persistent eosinophilia and high FeNO for the improvement of asthma control.

## Introduction

Asthma is a heterogeneous disease with different phenotypes 1. Recently, studies have been identifying different phenotypes defined by characteristic clinical manifestations, pathophysiological mechanisms, and biomarkers. Fractional exhaled nitric oxide (FeNO) and blood eosinophils have been used to distinguish patients with high T helper 2 (Type‐2) inflammation and to predict the therapeutic response to treatments targeting Type‐2 associated cytokines 1. Positive correlations were found between the FeNO levels and blood eosinophils in treated asthmatics 2. However, several previous studies have shown distinct associations between the FeNO levels or blood eosinophils and the clinical outcomes in terms of lung function, asthma control, and exacerbations 3, 4. Little is known about the relationships between the FeNO levels and blood eosinophils in asthmatics.

We have previously assessed the response to high dose systemic corticosteroids in terms of asthma control, lung function, blood eosinophils, and FeNO level in severe asthma patients with persistently high levels of FeNO despite maintenance therapy including high‐daily‐dose inhaled corticosteroids (ICS) 5. In this subanalysis, we investigated the responsiveness of FeNO and blood eosinophils to systemic corticosteroids treatment, and the change in Asthma Control Questionnaire (ACQ) 6 according to difference in steroid‐responsiveness of FeNO and blood eosinophils.

## Materials and Methods

Twenty severe asthmatics with persistent FeNO elevations (≥40 ppb) and blood eosinophilia (≥3%) despite maintenance therapy including high‐daily‐dose ICS (mean equivalent dose of 1760 µg beclomethasone dipropionate/day) were analyzed. All subjects were recruited from Wakayama Medical University Hospital. Long‐acting beta2‐agonist, leukotriene receptor antagonist, and theophylline were used to 20 (100%), 13 (65%), and 11 (55%) patients, respectively. ACQ, lung function test, blood eosinophils, and FeNO were assessed before and after 14 days treatment with 0.5 mg/kg oral prednisolone/day. The baseline mean values of the ACQ score, FeNO, and blood eosinophil percentage were 1.8, 60.1 ppb, and 8.2%. The study design and population characteristics have been previously reported in detail 5. This study was approved by the ethics committee of Wakayama Medical University (IRB #526) and registered with the University Hospital Medical Information Network (UMIN 000008401). Informed written consent was obtained from each participant.

In evaluating the responses of FeNO and blood eosinophils to systemic corticosteroids treatment, Spearman's correlation analysis was performed. The patients were divided into four groups based on the reduction in FeNO and blood eosinophils by systemic corticosteroid treatment as follows: steroid response (both ≥20% reduction in FeNO levels and blood eosinophils); poor response (FeNO) (<20% reduction in FeNO levels and ≥20% reduction in blood eosinophils); poor response (Eos) (≥20% reduction in FeNO levels and <20% reduction in blood eosinophils); poor response (FeNO and Eos) (both <20% reduction in FeNO levels and blood eosinophils).

We evaluated the differences in the ACQ score among the four groups in response to systemic corticosteroids treatment by Kruskal–Wallis test and the differences in the ACQ score between the steroid response group and poor response group by Mann–Whitney *U*‐test. The Kruskal–Wallis test, Mann–Whitney *U*‐test, and chi‐squared test were used to determine statistically significant differences in the patient characteristics.

## Results

**Figure 1:**
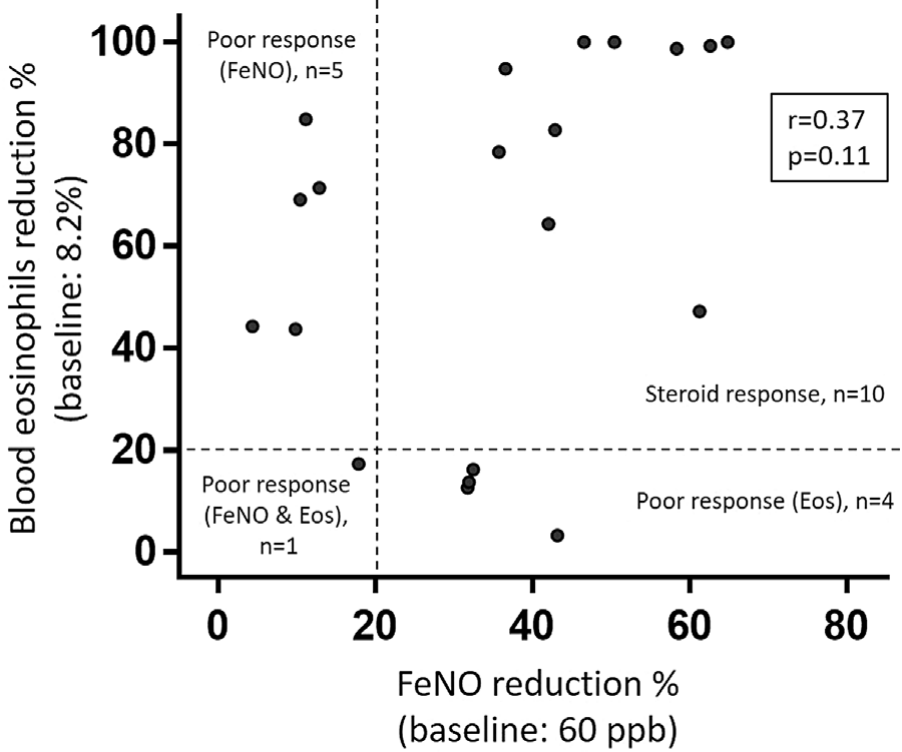
Variability of responses to systemic corticosteroids in FeNO and blood eosinophils. The patients were divided into four groups based on the reduction in FeNO and blood eosinophils by systemic corticosteroid treatment.

**Figure 2:**
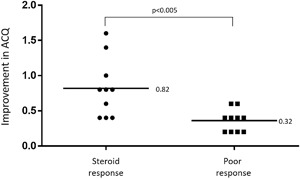
Changes in ACQ score according to differences in the steroid‐responsiveness of inflammatory biomarkers. There were significant differences in the ACQ score between steroid response group and poor response group (*P* < 0.005).

Twenty asthmatics were with persistent FENO elevation (≥40 ppb) and blood eosinophilia (≥3%) even after treatment with systemic steroid. As shown in Fig. 1, the changes by systemic steroid therapy in the blood eosinophils were not correlated with the changes in the FeNO levels (*r* = 0.37, *P* = 0.11). The steroid response group consisted of 10 patients (50%). There were no differences of patient's baseline characteristics between four groups (all *P* > 0.05, Table 1). There were significant differences in the ACQ score between the steroid response group and poor response group (*P* < 0.005, Fig. 2), while the percentage increase in FEV1 was close to significance (*P* = 0.074, data not shown). And among the four groups, only the steroid response group achieved a change in score of ≥0.5 on the ACQ (data not shown).

**Table 1:** Baseline characteristics of study patients subdivided by steroid response

	Steroid response (*n* = 10)	Poor response (FeNO) (*n* = 5)	Poor response (Eos) (*n* = 4)	Poor response (FeNO & Eos) (*n* = 1)
Age (years)	53 (29–69)	52 (34–62)	61 (41–68)	60
Gender (female/male), *n*	5/5	2/3	2/2	1/0
Body mass index (kg/mm^2^)	22.7 (17.3–29.4)	23.3 (17.3–28.3)	23.0 (18.8–30.1)	18.0
Smoking status (never/ex), *n*	5/5	1/4	2/2	1/0
Atopy, *n* (%)	9 (90)	5 (100)	3 (75)	1 (100)
Duration of asthma (years)	15 (5–36)	17 (12–31)	17 (9–26)	29
Allergic rhinitis, *n* (%)	9 (90)	5 (100)	3 (75)	0 (0)
Chronic rhinosinusitis, *n* (%)	4 (40)	1 (25)	1 (25)	0 (0)
FVC (L)	2.98 (1.43–4.96)	3.34 (3.00–4.81)	3.12 (1.76–4.22)	2.79
FVC % of predicted (%)	90.7 (69.1–95.7)	91.3 (76.1–102.3)	88.5 (75.0–94.3)	97.7
FEV_1_ (L)	1.71 (0.64–2.87)	2.35 (1.31–3.20)	2.49 (1.19–3.39)	1.73
FEV_1_/FVC ratio (%)	56.5 (43.3–81.5)	66.5 (43.4–78.3)	77.0 (67.6–87.8)	62.0
FEV_1_ % of predicted (%)	55.9 (46.6–79.2)	74.3 (37.2–78.0)	75.1 (63.8–79.8)	78.8
Inhaled corticosteroids, *n* (%)	10 (100)	5 (100)	4 (100)	1 (100)
Dose of inhaled corticosteroids (µg/day)^a^	900 (800–1000)	800 (800–1000)	800 (800–1000)	1000
Inhaled long‐acting β_2_‐agonist, *n* (%)	10 (100)	5 (100)	4 (100)	1 (100)
Leukotriene receptor antagonist, *n* (%)	9 (90)	2 (40)	2 (50)	0 (0)
Blood eosinophil counts (cells/mL)	487 (390–1464)	416 (233–695)	509 (192–796)	293
Exhaled nitric oxide fraction (ppb)	57.2 (40.5–89.2)	45.2 (40.9–93.5)	45.8 (41.3–105.4)	52.2
Serum total immunoglobulin E (IU/mL)	574 (76–2568)	301 (80–2878)	151 (32–390)	38

Values represent the median (min–max), unless otherwise stated.

^a^ Inhaled corticosteroids, expressed as beclomethasone dipropionate equivalent.

## Discussion

Our study revealed two important clinical findings. First, in the patients with severe asthma there was variability in the responses to systemic corticosteroids in FeNO and blood eosinophils. The reason for this might be pathophysiologic differences between these two biomarkers. Type‐2 driven cytokines IL‐4, IL‐5, IL‐13 are associated with systemic and airway inflammation in asthma 1. IL‐5 is responsible for eosinophilic inflammation and it is a specific and strong stimulus for eosinophil differentiation 7. FeNO is a useful surrogate marker for assessing airway inflammation, primarily that triggered by IL‐4 and IL‐13 in the bronchial mucosa 8. The measurement of blood eosinophils is widely used in clinical practice when monitoring asthmatic patients, and eosinophilia is primarily triggered by IL‐5 7, whereas the IL4/IL‐13 pathway does not seem to affect the blood eosinophils 8. In our study, the steroid response group may reflect the suppression of both Type‐2 cytokines such as IL‐5 and IL‐4/IL‐13. The poor response (FeNO) group may reflect the suppression of only suchType‐2 cytokines as IL‐5, and the poor response (Eos) group may reflect the suppression of only such Type‐2 cytokines as IL‐4/IL‐13. Molecular mechanisms of severe asthma that can be suppressed by systemic steroids was shown to be different depending on cases.

Second, it was necessary to suppress both persistent eosinophilia and high FeNO for the improvement of asthma control. Interestingly, there is one study that analyzed the association between a combination of increased levels of FeNO, a local biomarker of Type‐2 inflammation, and blood eosinophils, a systemic Type‐2 marker, and asthma morbidity in a clinical study of patients with asthma 9. Simultaneously increased FeNO and blood eosinophils were related to a higher likelihood of bronchial hyperresponsiveness and uncontrolled asthma in a large cohort of young asthmatic patients. Our results may indicate that suppression of either IL‐5 or IL‐4/IL13 is insufficient and it is necessary to suppress both Type‐2 cytokines such as IL‐5 and IL‐4/IL‐13 for the improvement of asthma control.

In the present study, 50% of the patients with severe asthma could not achieve suppression of either or both FeNO and blood eosinophils even after treatment with systemic steroid. Furthermore, 30% of the patinets with steroid rensponse group could not achieved a change in score of ≥0.5 on the ACQ. To investigate the factor that improve the ACQ score in the steroid response group, we compared the baseline characteristics between ACQ responders (*n* = 7) and non‐responders (*n* = 3). Drug treatment, lung function, FeNO levels, and blood eosinophil numbers were similar between the groups. The non‐responders were all ex‐smokers, whereas in responders there were as many as five never‐smokers (*P* = 0.038, data not shown). Thus, smoking status might be influenced the change of symptom in the steroid response group. These results suggest that they are steroid‐resistant asthma patients who may need different treatment such as molecularly targeted therapies. Many antagonists of Type‐2 cytokines are under development as eosinophilic asthma therapeutics 1, so it has become more necessary to find the best companion diagnostics test for such targeted therapies. Capturing the trend of Type‐2 cytokines such as IL‐4/IL‐13 and IL‐5, our systemic steroid trial may be applicable to companion diagnostics testing for molecularly targeted therapies.

There would be some limitations in our study. First, the sample size was relatively small. Second, this study was an extended analysis from a previously published uncontrolled trial. Third, blood eosinophilia was not expressed in absolute numbers, since systemic corticosteroids might affect other leukocytes subsets besides blood eosinophils.

In summary, in the severe asthma patients, there was variability in the responses to systemic corticosteroids in FeNO and blood eosinophils. It is essential to suppress both FeNO and blood eosinophils for the improvement of asthma control.

## Conflict of Interest

The authors declare no conflict of interests.
